# Lumbar spinal stenosis and disc alterations affect the upper lumbar spine in adults with achondroplasia

**DOI:** 10.1038/s41598-020-61704-w

**Published:** 2020-03-13

**Authors:** Thomas Huet, Martine Cohen-Solal, Jean-Denis Laredo, Corinne Collet, Geneviève Baujat, Valérie Cormier-Daire, Alain Yelnik, Philippe Orcel, Johann Beaudreuil

**Affiliations:** 1 Université de Paris, BIOSCAR Inserm U1132 and Department of Rheumatology and Reference Center for Constitutional Bone Diseases, AP-HP Hospital Lariboisière, F-75010 Paris, France; 2 Université de Paris, Department of Bone and Joint Imaging, AP-HP Hospital Lariboisière, F-75010 Paris, France; 3 Université de Paris, Department of Biochemistry and Genetics, AP-HP Hospital Lariboisière, F-75010 Paris, France; 4 Université de Paris, Department of Genetics, Reference Center for Constitutional Bone Diseases, AP-HP Hospital Necker, Paris, France; 5 Université de Paris, Department of Physical Medicine and Rehabilitation, AP-HP Hospital Fernand Widal, Paris, France

**Keywords:** Genetics, Skeleton

## Abstract

In achondroplasia, lumbar spinal stenosis arises from congenital dysplasia and acquired degenerative changes. We here aimed to describe the changes of the lumbar spinal canal and intervertebral disc in adults. We included 18 adults (age ≥ 18 years) with achondroplasia and lumbar spinal stenosis. Radiographs were used to analyze spinal-pelvic angles. Antero-posterior diameter of the spinal canal and the grade of disc degeneration were measured by MRI. Antero-posterior diameters of the spinal canal differed by spinal level (P < 0.05), with lower values observed at T12-L1, L1-2 and L2-3. Degrees of disc degeneration differed by intervertebral level, with higher degrees observed at L1-2, L2-3 and L3-4. A significant correlation was found between disc degeneration and thoraco-lumbar kyphosis at L2-3, between antero-posterior diameter of the spinal canal and lumbar lordosis at T12-L1 and L2-3, and between antero-posterior diameter of the spinal canal and thoraco-lumbar kyphosis at L1-2. Unlike the general population, spinal stenosis and disc degeneration involve the upper part of the lumbar spine in adults with achondroplasia, associated with thoraco-lumbar kyphosis and loss of lumbar lordosis.

## Introduction

Achondroplasia is the most common form of skeletal dysplasia with micromelia, with an estimated prevalence is 1/16,000–25,000 live births^1,2^. The disease is due to a gain-of-function mutation in the fibroblast growth factor receptor 3, that affects growth through chondrocyte dysfunction and cartilage dysplasia^3,4^. The clinical consequence is a disproportionate short stature with short limbs and cranial and spine abnormalities^1,2^. Spinal disorders are major contributors to disability in adults with achondroplasia^5^. They include cervico-medullary compression and spinal stenosis, this being the most prevalent spinal feature of this condition. Approximatively 40% of adults with achondroplasia have chronic back problems. Half undergo surgery because of lumbar spinal stenosis^6^.

The clinical expression of lumbar spinal stenosis ranges from low back pain with neurogenic claudication to paraplegia with bladder and bowel dysfunction^7^. Achondroplasia-associated lumbar spinal stenosis results from a combination of congenital dysplasia and acquired degenerative changes^8^. Spinal dysplasia in achondroplasia is due to disordered endochondral ossification resulting in early fusion of the pedicles to the vertebral body^9^. Hence the cross-sectional area of the spinal canal is narrowed by the shortened pedicles and the decreased interpedicular distance. The dimensions of the spinal canal are further reduced by age-related degenerative changes, which eventually compromise the space available for the neural elements.

Lumbar spinal stenosis is also found in individuals without dysplasia^10–12^. The occurrence of the stenosis increases with aging. Acquired degenerative changes involved in lumbar spinal stenosis have largely been described in general populations, but less so in adults with achondroplasia^8,13^. The degenerative process of lumbar spinal stenosis leads to disc narrowing and bulging, infolding and thickening of the ligamentum flavum as well as facet osteoarthritis^10–12^. With advanced alterations, disc, ligamentum flavum, and facet joints protrude into the spinal canal. Increased stresses associated with disc degeneration and narrowing in turn induce in turn facet-joint and ligamentum-flavum degeneration. Thus degenerative disc changes may be a critical step in the cascade of events leading to lumbar spinal stenosis with its clinical manifestations.

Degenerative changes of the intervertebral disc have not been characterized in adults with achondroplasia. The identification of current changes is important to assess the long term impact of future therapies. Therefore, we conducted a retrospective study of achondroplasia patients with lumbar spinal stenosis in a single center to characterize degenerative changes of the intervertebral disc in this population and to identify factors associated with the process.

## Results

### Patient characteristics and measures

Eighteen adults with achondroplasia were referred to the reference center for constitutional bone diseases between January 2004 and February 2017, fulfilled the inclusion criteria and were included in the study. All had back and leg symptoms attributed to lumbar spinal stenosis. Clinical characteristics of included patients are in Table 1. Two patients had previously undergone lumbar laminectomy, with five (T12-L5) and three (L2-L5) operated levels. Each of the eight operated levels was excluded from the imaging analysis of antero-posterior diameter and disc degeneration. A total of 14 patients including one with previous spinal surgery, underwent spine radiographies and spino-pelvic angle assessment. Reliability of measurements was good to very good. Intra- and inter-observer ICCs were 0.93 (95% CI 0.86–0.96) and 0.92 (0.84 0.96) respectively for measuring antero-posterior spinal canal diameter. Intra- and inter-reliability kappa values were 0.87 (95%CI 0.73–1) and 0.78 (0.61–0.95) for measuring disc degeneration with the Pfirmann grading system.

**Table 1:** Clinical characteristics and radiographic spino-pelvic angles of the study population.

Clinical characteristics (n = 18)	
Age, years, mean (SD)	37 (17)
Women, n (%)	11 (61)
Height, cm, mean (SD)	127 (7)
Weight, kg, mean (SD)	57 (13)
Body mass index, kg/m^2^, mean (SD)	35 (8)
**Radiographic spino-pelvic angles in degrees (n = 14)**	
Sacral slope, mean (SD)	43 (7)
Pelvic tilt, mean (SD)	7 (12)
Pelvic incidence, mean (SD)	50 (13)
Lumbar lordosis, mean (SD)	46 (11)
Thoraco-lumbar kyphosis, mean (SD)	7 (8)

### Spinal stenosis and disc alterations affect mainly the upper lumbar spine

**Figure 1:**
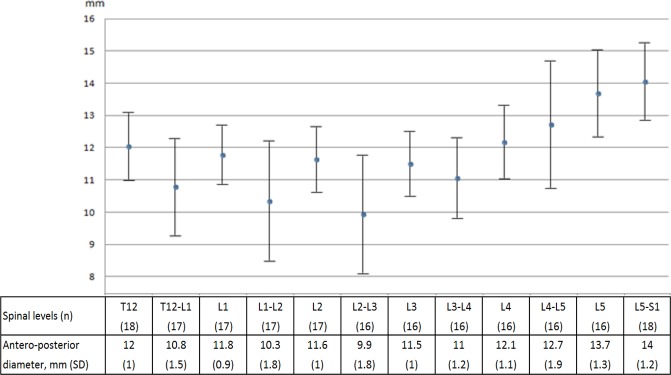
Values of antero-posterior diameter of the spinal canal. Difference between spinal levels, Kruskall-Wallis test, P = 0.0001.

**Figure 2:**
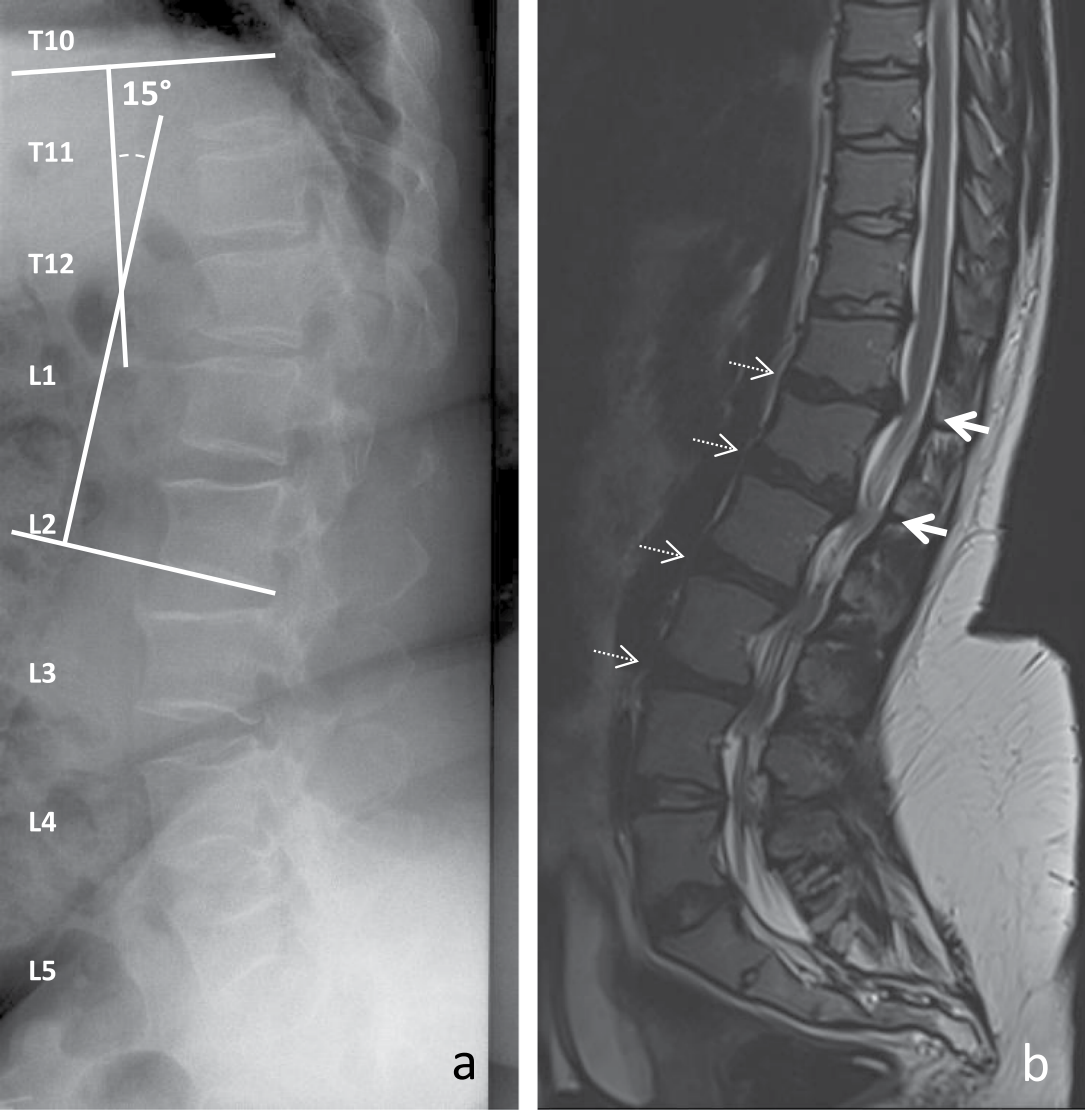
Representative lumbar spine imaging in adults with achondroplasia. (**a**) X-ray of a 58 year-old women revealing a thoraco-lumbar kyphosis. (**b**) T2-weighted MRI of a 25 year-old women showing an involvement of the upper part of the lumbar spine including T12-L2 antero-posterior stenoses (thick arrows) and T12-L4 disc degeneration (dark discs, dashed arrows).

Considering the study population, antero-posterior diameters of the spinal canal differed by vertebral or intervertebral disc level (Fig. 1), with lower values observed at T12-L1, L1-2 and L2-3 intervertebral levels. Lumbar spinal stenosis was maximal at L2-3. In addition, degrees of disc degeneration differed by intervertebral disc level (Tables 2 and 3), with higher degrees observed at L1-2, L2-3 and L3-4. The most advanced degenerative changes were again observed at L2-3. The involvement of the upper lumbar spine is illustrated in Fig. 2.

**Table 2:** Assessment of disc degeneration with the Pfirrman grading system^23^.

Spine levels	Grade 1	Grade 2	Grade 3	Grade 4	Grade 5
T12-L1	12 (71)	1 (6)	2 (12)	1 (6)	1 (6)
L1-L2	9 (53)	2 (12)	1 (6)	4 (24)	1 (6)
L2-L3	5 (31)	1 (6)	3 (19)	7 (44)	0 (0)
L3-L4	4 (25)	3 (19)	6 (38)	3 (19)	0 (0)
L4-L5	7 (44)	7 (44)	1 (6)	1 (6)	0 (0)
L5-S1	4 (22)	12 (67)	1 (6)	1 (6)	0 (0)

Data are n (%).

Grade 1, homogeneous disc with bright high signal intensity, clear distinction between nucleus and annulus, and normal disc height.

Grade 2, inhomogeneous disc with white signal intensity, clear distinction between nucleus and annulus, and normal height.

Grade 3, inhomogeneous disc with intermediate gray signal intensity, unclear distinction between nucleus and annulus, and normal or slightly decreased disc height.

Grade 4, inhomogeneous disc with dark low signal intensity, no possible distinction between nucleus and annulus, and normal or moderately decreased disc height.

Grade 5, inhomogeneous disc with black low signal intensity, no possible distinction between nucleus and annulus, and complete disc narrowing.

**Table 3:** Pfirrmann scores of disc degeneration^23^.

Levels (n)	Scores
T12-L1 (17)	1.7 (1.3)
L1-L2 (17)	2.2 (1.5)
L2-L3 (16)	2.8 (1.3)
L3-L4 (16)	2.5 (1.1)
L4-L5 (16)	1.8 (0.9)
L5-S1 (18)	1.9 (0.7)

Data are mean (SD).

Difference between spinal levels, Kruskall-Wallis, P = 0.0001.

Correlation analysis of disc degeneration and clinical parameters indicated a significant correlation with only age at the L2-3 (r_s_=0.50), L3-4 (r_s_=0.58), L4-5 (r_s_=0.78) and L5-S1 (r_s_=0.69, p < 0.05), and with height (r_s_ = −0.55, p < 0.05) at L4-5. Correlation analysis of antero-posterior diameter of the spinal canal and clinical parameters indicated no significant correlation from T12-L1 to L5-S1.

For the radiographic spino-pelvic angles investigated, we found a significant correlation between disc degeneration and thoraco-lumbar kyphosis at L2-3 (Table 4), between antero-posterior diameter of the spinal canal and lumbar lordosis at T12-L1 and L2-L3, and between antero-posterior diameter of the spinal canal and thoraco-lumbar kyphosis at L1-2 level (Table 5).

**Table 4:** Spearman correlation coefficients between disc degeneration and radiographic spino-pelvic angles (n = 14).

Spino-pelvic parameters	T12-L1	L1-L2	L2-L3	L3-L4	L4-L5	L5-S1
Sacral slope	0.139	−0.026	−0.176	0.202	0.075	−0.237
Pelvic tilt	−0.362	−0.198	0.122	0.066	0.014	−0.059
Pelvic incidence	−0.312	−0.159	−0.086	0.128	0.005	−0.288
Lumbar lordosis	−0.192	0.077	−0.321	0.235	0.128	−0.132
Thoraco-lumbar kyphosis	0.478	0.313	0.554*	0.291	0.268	0.203

*P < 0.05.

**Table 5:** Spearman correlation coefficients between antero-posterior diameter of the spinal canal and radiographic spino-pelvic angles (n = 14).

Spino-pelvic parameters	T12-L1	L1-L2	L2-L3	L3-L4	L4-L5	L5-S1
Sacral slope	0.189	−0.044	0.526	0.066	0.113	0.086
Pelvic tilt	0.321	0.023	−0.206	0.230	−0.262	−0.210
Pelvic incidence	0.387	0.008	0.151	0.179	−0.098	−0.001
Lumbar lordosis	0.659*	0.516	0.887*	0.410	−0.105	−0.014
Thoraco-lumbar kyphosis	−0.461	−0.547*	−0.355	−0.189	−0.367	−0.179

*P < 0.05.

We also did a secondary analysis excluding the patient with previous spinal surgery. All correlations persisted, with P value <0.05 for correlation between disc degeneration and thoraco-lumbar kyphosis at L2-3 as for correlation between antero-posterior diameter of the spinal canal and lumbar lordosis at T12-L1 and L2-L3; and with P value = 0.05 for correlation between antero-posterior diameter of the spinal canal and thoraco-lumbar kyphosis at L1-2 level.

## Discussion

Herein, we describe degenerative changes of the intervertebral disc in adults with achondroplasia. In this single-center series, spinal stenosis and disc degeneration mainly involved the upper part of the lumbar spine, with maximal changes at L2-3. The degree of spinal stenosis was not correlated with age, sex, height, weight or BMI. Disc degeneration was correlated with age and height. However, this relationship was more pronounced at lower lumbar intervertebral disc levels. In contrast to clinical factors, radiographic spino-pelvic angles were correlated with spinal canal diameters and disc changes at the upper, but not lower part of the lumbar spine. Thoraco-lumbar kyphosis was positively correlated with L2-3 disc degeneration, and both thoraco-lumbar kyphosis and loss of lumbar lordosis were correlated with spinal stenosis from T12-L1 to L2-3.

Distribution of spinal stenosis among intervertebral thoraco-lumbar levels in our achondroplasia cohort differs from the preferential lower lumbar-spine location in the general population, with maximal shrinking at L4-5 level^14^. Preferential involvement of stenosis in the upper lumbar spine in achondroplasia was previously reported^8,15^. The lowest values of the antero-posterior canal diameter were found at L2-3^8^. Furthermore, surgery for lumbar spinal stenosis in achondroplasia concerns L1-2, L2-3 and L3-4 in more than 50% of cases and the L2-3 in as much as 97% of cases^16^. Location of the highest degrees of disc degeneration at L1-2, L2-3 and L3-4 in our patients also contrasts with the highest prevalence at the lower lumbar level, especially L4-5, in the general population^17^. To our knowledge, the distribution of disc degeneration in adults with achondroplasia has not previously been reported.

To our knowledge, correlations between spinal stenosis or disc degeneration, and age, sex, weight, height or BMI have not been investigated in achondroplasia patients. We found a correlation between disc degeneration and age at L2-3, L3-4, L4-5 and height at L4-5. These findings agree with age-associated disc degeneration features in the general population. However, we found no association with other risk factors of disc degeneration identified in the general population, such as body weight and BMI^18,19^. Height was not related to disc degeneration in the general population. The correlation between disc degeneration and height in our patients should be viewed with caution. Its reasons appear difficult to describe. Conversely to age and disc degeneration, it involves L4-5 only and not higher levels which are more affected by disc degeneration; and no such association has previously been found in general population. The correlations between MRI findings and radiographic spino-pelvic parameters involved the upper part of the lumbar spine only and therefore are likely to be directly related to lumbar spinal stenosis. Even if not all statistically significant for each intervertebral disc level in the area of interest, our results suggest that the spino-pelvic angles, which characterize developmental spinal dysplasia, may have a role in the pathogenesis of disc degeneration and spinal stenosis at the upper lumbar spine in adults with achondroplasia. The results support the hypothesis that thoraco-lumbar kypkosis is a factor of disc degeneration and spinal stenosis. Arguments for this hypothesis are as follows: thoraco-lumbar kyphosis is associated with disc degeneration and spinal stenosis in our patients; previous biomechanical data in human suggest that kyphosis increases forces acting on discs which can contribute to disc degeneration and spinal stenosis^20^; clinical manifestations usually attributed to disc degeneration or spinal stenosis have been reported to be associated with thoraco-lumbar kyphosis in adults with achondroplasia^21,22^. We also found loss of lumbar lordosis related to thoraco-lumbar spinal stenosis. Loss of lumbar lordosis was previously found associated with pain in adults with achondroplasia^14^. However our results involving lumbar lordosis should be cautiously considered. Indeed, we assessed thoraco-lumbar kyphosis from T10 to L2, and lumbar lordosis from L1 to S1. Due to the overlap of the measure, the values of the thoraco-lumbar kyphosis may have influenced the correlations involving lumbar lordosis. Further investigations are therefore required to separately assess lordosis in upper and in lower levels of the lumbar spine. They may also be useful to clarify the association of thoraco-lumbar kyphosis with disc degeneration and spinal stenosis.

Our study provides some insight into associations among disc degeneration, lumbar spinal stenosis and radiographic spino-pelvic parameters in achondroplasia. However, it is limited by its small sample size, retrospective design, no quantitative assessment of pain and functional limitation, no pain-free control, and no follow-up. Furthermore, the radiographic spino-pelvic analysis did not involve the full spine. Data on lumbar spine in adults with achondroplasia are sparse and analysis of 18 patients is of interest in this context. The small sample we used was due to the rare disease and close to that of others^8^.

In conclusion, we describe lumbar spinal stenosis and disc degeneration in adults with achondroplasia. Disc degeneration was not previously described in this population. Our results suggest that spinal stenosis and disc degeneration are located at the upper part of the lumbar spine, with maximal changes at L2-L3. We also suggest their association with thoraco-lumbar kyphosis and loss of lumbar lordosis. Further investigations remain necessary to reinforce our results and hypotheses, and for better understanding of the condition.

## Patients and methods

The study was a retrospective case series. Due to the rare disease, the sample size was not planned, but convenience sample. Adults (age ≥ 18 years) with achondroplasia referred to the reference center of constitutional bone diseases between January 2004 and February 2017 were considered for inclusion. Patients with available MR images of the lumbar spine were included in the study. The study involved review of clinical information by the treating clinicians. It does not require ethics approval. No patient expressed any opposition to the study.

### Clinical and imaging parameters

Back and leg symptoms, age, sex, height and body mass index (BMI) were recorded from medical charts. Radiographs of the lumbar spine, thoraco-lumbar junction, and hip joints in standing position, were analyzed. Spino-pelvic angles measured on these radiographs included the sacral slope (SS), pelvic tilt (PT), pelvic incidence (PI), lumbar lordosis (LL) and thoraco-lombar kyphosis (TLK)^21^. The SS corresponded to the angle between the sacral endplate and the horizontal plane; the PT, the angle between the line joining the middle of the sacral endplate and the hip axis, and the vertical plane; the PI, the angle between a line perpendicular to the sacral endplate and a line joining the middle of the sacral plate and the hip axis; the LL, the angle between the upper endplate of L1 and the upper endplate of S1; and the TLK, the angle between the upper endplate of T10 and the lower endplate L2.

T1-weighted and T2-weighetd sequences MR imaging sequences of the lumbar spine were obtained with a 1.5-T MR system. The antero-posterior diameters of the spinal canal were measured on mid-sagittal T2-weighted MR images. The antero-posterior diameter of the spinal canal was measured at each mid-height of the disc space from T12 to S1, and at each mid-vertebral level from T12 to L5. The anterior and posterior margins of the thecal sac were used as anterior and posterior landmarks of the spinal canal^8^. In case of previous lumbar spinal surgery, operated levels were excluded from the analyses. Measurements on radiographs and MR images involved using a Vue PACS workstation (v11.3, Carestream Health, Rochester, NY).

Mid-sagittal T2-weighted MR images were used for grading degenerative intervertebral disc changes. Degenerative intervertebral disc changes at each intervertebral disc level of the lumbar spine were assessed with the Pfirrmann grading system^23^. The Pfirrmann grading system involves five degrees from normal disc to advanced degeneration: grade 1, homogeneous disc with a bright high signal intensity, clear distinction between nucleus and annulus, and normal disc height; grade 2, inhomogeneous disc with a white signal intensity, clear distinction between nucleus and annulus, and normal height; grade 3, inhomogeneous disc with an intermediate gray signal intensity, unclear distinction between nucleus and annulus, and normal or slightly decreased disc height; grade 4, inhomogeneous disc with a dark low signal intensity, no possible distinction between nucleus and annulus, and normal or moderately decreased disc height; and grade 5, inhomogeneous disc with black low signal intensity, no possible distinction between nucleus and annulus, and complete disc narrowing. Each intervertebral disc was given a score from 1 to 5 according to the degrees of degeneration by the Pfirrmann grading system.

### Statistical analysis

To ensure the study results, measurements of the antero-posterior diameter of the spinal canal and degenerative intervertebral disc changes, were assessed for reliability. In total, 30 measurements of the canal diameter and disc degeneration were tested in the study population. Two independent physicians specialized in spinal disorders participated in the testing. One (TH) measured imaging parameters twice at a 3-month interval. The other (JB) measured parameters once. Intra- and inter-observer reliability was expressed by the intra-class correlation coefficient (ICC) for canal diameter and kappa coefficient for disc degeneration. Reliability results were given with 95% confidence intervals (95%CIs). Reliability was considered poor with values <0.41, moderate ≥ 0.41, good ≥ 0.61, and very good ≥ 0.81^24^. All reported parameters in the study were further measured by the same investigator (TH). Quantitative variables are described with mean ± SD. Categorical variables are described with number (%). Comparisons involved the Kruskal-Wallis test. Correlation was assessed with the Spearman correlation coefficient. Statistical significance was set at P < 0.05. All statistical analyses involved using XLSTAT (Addinsoft, France).

## Data Availability

The authors state that the data will be available to readers for non-profit companies upon request.
